# A comparative study of fluoride release from two different sealants

**DOI:** 10.4317/jced.51507

**Published:** 2014-12-01

**Authors:** Shimoga-Raju Ananda, Halappa Mythri

**Affiliations:** 1 Senior Lecturer. Department of Community Dentistry, Malabar Dental College and Research Centre, Mudur (po), Edappal, India; 2 Senior lecturer. Department of Public Health Dentistry, Sri Siddhartha Dental College, Tumkur, Karnataka, India

## Abstract

Objectives: The introduction of fluoride releasing sealants and glass ionomer cements as fissure sealants adds another dimension to prevention of pit and fissure caries. The ability of resin sealants and glass ionomer cements to release fluoride on a long term basis to the sealed enamel and the adjacent unsealed pit and fissure and cuspal incline enamel may allow for further reduction in pit and fissure caries experience for children. Hence, the study was conducted to compare the amount of fluoride release in the plaque after placing fluoride releasing pit and fissure sealants and glass ionomer fissure sealants used in Atraumatic Restorative Treatment (ART) approach. To compare the fluoride release of both the materials at the different time intervals. Material and Methods: A total of 60 school going children were included in this study. Before application of the sealants, baseline plaque fluoride levels were estimated from all the study subjects. After application of sealants again the same was estimated at an interval of 24 hour, 9 days, 2 weeks and 4 weeks. Results: The peak plaque fluoride levels were achieved at 24 hours after application of fissure sealants in all the groups. Conclusions: Within the limitation of the study, the present study indicated that fluoride releasing fissure sealants may act as a source of fluoride in plaque which will help in preventing pit and fissure and smooth surface caries in the tooth sealed with fissure sealants.

** Key words:**Plaque fluoride, pit and fissures sealants, dental caries.

## Introduction

To prevent pit and fissure caries the concept of altering pit and fissure morphology as a means of reducing the susceptibility has been in vogue for over 100 years (1). Following the description of the acid – etch technique by Buonocore (2), numerous studies have reported on the effectiveness of fissure sealants as caries preventive agents (3,4) and at present, sealing with resin based pit and fissure sealant materials is a recommended procedure to prevent caries on the occlusal surfaces of permanent molars (5).

American Dental Association [ADA] accepted pit and fissure sealants in 1971 (6) and since then pit and fissure sealants have experienced a series of modifications in the materials use (7) and application techniques (8). Introduction of fluoride releasing sealants adds another dimension.

Fissure sealing with glass ionomer cement was introduced in 1974 by Mclean and Wilson (9). Glass-Ionomer Cements [GICs] are known to release fluoride ions into the oral cavity. Several invitro and insitu studies have also shown that release of fluoride either in saliva or dental plaque from glass ionomer materials is thought to protect the tooth against dental caries. This benefit of fluoride release and subsequent adsorption is found not only in enamel immediately adjacent to glass ionomer restorations, but also in areas up to 3 mm away from the restoration margins and may even protect the entire tooth (10).

With the advent of new glass ionomer sealant [Fuji IX and Ketac Molar] which claims to be possessing better properties, it becomes necessary to compare and evaluate these fissure sealants with fluoride releasing methacrylate sealants for their clinical efficacy in releasing fluoride to the dental plaque in the adjacent tooth structure where sealant has been applied.

- Aim and objectives:

1. To compare the amount of fluoride release in plaque after placing fluoride releasing pit and fissure sealants and glass ionomer fissure sealants.

2. To compare the fluoride release from both the materials in plaque at different time intervals.

## Material and Methods

The present comparative in vivo clinical trial was conducted on a total of 60 school going children at Davangere. Necessary Ethical Clearance and official permission was obtained.

- Materials used: Four fissure sealants were used for the study and are randomly divided into 4 groups by lottery method with 15 subjects in each group.

1. Teethmate-F1 [Methacryloyl methyl methacralate containing sealant] - Group I 

2. Helioseal-F [sealant containing fluorosilicate glass] - Group II

3. Fuji IX GP [high-viscosity glass ionomers used in Atraumatic Restorative Treatment] - Group III

4. Ketac-Molar [high-viscosity glass ionomers used in Atraumatic Restorative Treatment] - Group IV

## Sample Collection

The subjects were provided with non-fluoridated dentifrice during experimental period and were asked to refrain from all other oral hygiene procedures during the study duration, and instructed to restrict the intake of foods containing high fluoride content and abstain from drinking tea. Baseline plaque fluoride levels, salivary pH and DMFT/ def index were estimated.

To obtain baseline values subjects were instructed not to brush their teeth on the morning of sample collection day. Plaque samples were collected from all the subjects on three consecutive days and mean was taken. It was collected from accessible surfaces of first permanent molar in all quadrants using curette instrument.

The same procedure of plaque was followed after application of sealants at an interval of 24 hour, 9 days, 2 weeks and 4 weeks after application of sealants.

- Fluoride analysis: The plaque samples were incubated for 3 hours at 370c in the presence of phosphatase enzyme in order to hydrolyze any monofluorophosphate [FPO32-] ions to F. 0.1 ml of 5U / L of sodium acetate buffer [pH 4.8] was added to 1 ml of saliva sample. Fluoride ion activity was then measured in the presence of TISAB buffer with fluoride ion specific electrode Orion 94-09 (11).

- Statistical analysis: Mean and standard deviations for the 4 samples from each material at various interval of time were determined. Data collected by experiments were computerized and analyzed using the Statistical Package for the Social Sciences [SPSS version 17.0].

Mean and Standard Deviation [SD] were calculated. One way Analysis Of Variance [ANOVA] test was used for multiple group comparisons followed by Tukeys post hoc for group wise comparisons. An unpaired student t test was used to compare two materials. *P*-value <0.05 were considered statistically significant.

## Results

The present study was carried out to evaluate the fluoride release into dental from 4 fluoride releasing fissure sealants [2 pit and fissure sealants and two glass ionomer cements used as fissure sealants] at different intervals of time i.e. after 24 hours, 9 days, 2 weeks and 4 weeks after application of sealants.

The amount of fluoride release into dental plaque was measured in Bapuji Institute of Engineering College Davangere.

 Table 1 reveals the mean salivary pH and dmfs of subjects under different group. ANOVA reveled the salivary pH at baseline was not statistically significant between the groups with a *p* value 0.10.

**Table 1:**
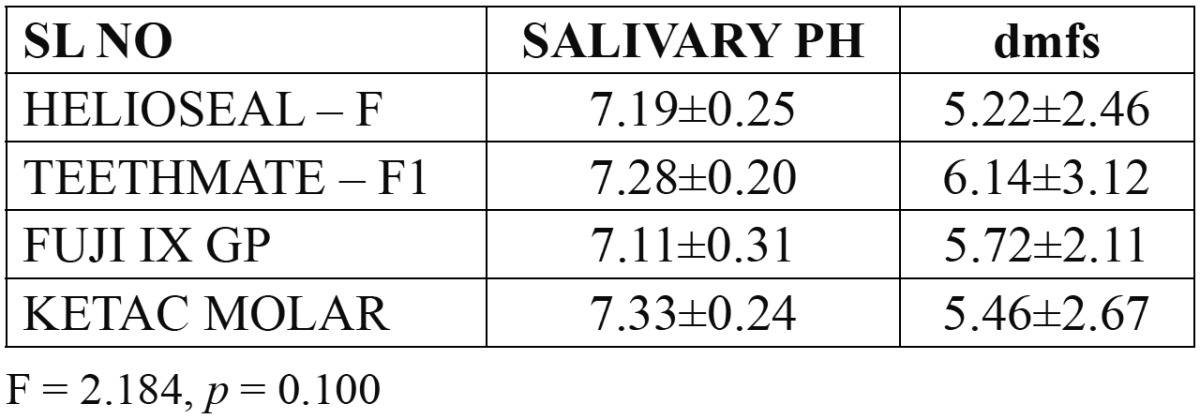
Mean salivary pH and dmfs of subjects under different group.

 Table 2 shows the mean baseline plaque fluoride levels were 32.79±2.32, 33.42±3.31, 31.93±4.68 and 32.85±3.98 for group I to group IV respectively. An Analysis of Variance test [ANOVA] revealed that there was no statistically significant difference between different groups.

**Table 2:**
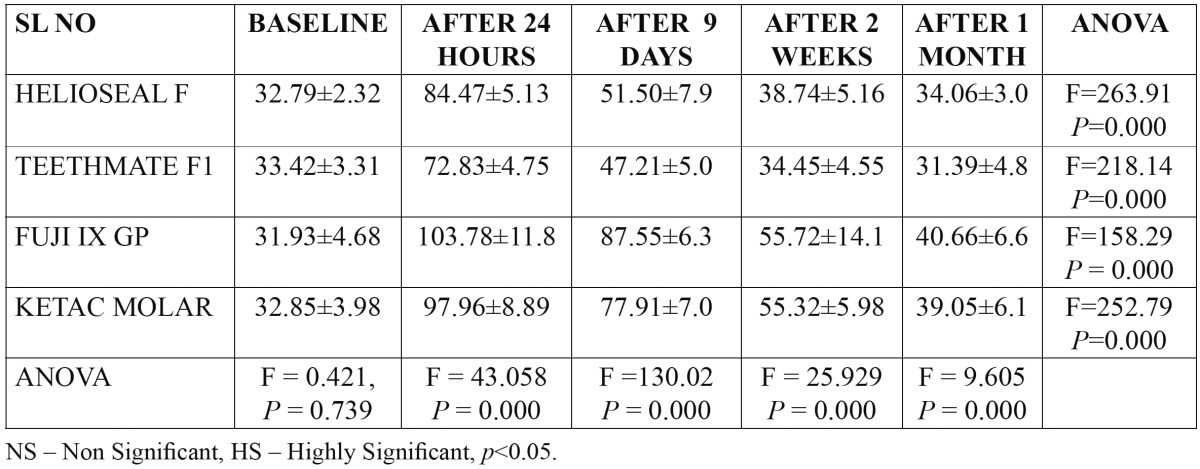
Mean plaque fluoride levels of subjects at different time intervals between & within different materials.

The peak plaque fluoride level were achieved 24 hours after application of fissure sealants in all the groups i.e. 84.47±5.13, 72.83±4.75, 103.78±11.86 and 92.63±24.11 for group I to group IV respectively and an analysis of variance [ANOVA] reveled a statistically significant difference between different fissure sealants [*p*<0.000] with the Post Hoc Tukeys test showing statistical difference between Helioseal and Teethmate F1, Fuji IX and Helioseal F, Fuji IX and Teethmate F1, Ketac Molar and Helioseal F and Ketac Molar and Teethmate F1. With Fuji IX fissure sealant showing highest plaque fluoride levels.

Determined at 9 days interval again there was a statistical significant difference between Fuji IX and Helioseal F, Fuji IX and Teethmate F1, Fuji IX and Ketac Molar, Ketac Molar and Helioseal F and Ketac Molar and Teethmate F1.

Determined at 2 weeks interval the statistical significant difference was found between Fuji IX and Helioseal F, Fuji IX and Teethmate F1, Ketac Molar and Helioseal F and Ketac Molar and Teethmate F1. And at the end of the study statistically significant difference was found between Fuji IX and Helioseal F, Fuji IX and Teethmate F1 and Ketac Molar and Teethmate F1.

Among the two fissure sealants tested i.e. Helioseal F and Teethmate F1, the salivary fluoride levels were not statistically significant at any of the intervals whereas the plaque fluoride levels differed significantly at 24 hours after application of fissure sealants, determined at 9 days, 2 weeks and 4 weeks, the levels were not statistically significant.

Among the two glass ionomer fissure sealants tested i.e. Fuji IX and Ketac Molar the salivary fluoride levels were statistically significant at 24 hours after application of fissure sealants determined at 9 days, 2 weeks and 4 weeks, the levels were not statistically significant.

Whereas the plaque fluoride levels differed significantly at 9 days after application of fissure sealants determined at 24 hours, 2 weeks and 4 weeks the levels were not statistically significant.

 Table 2 also shows comparison within the material at various intervals which revealed the following result. Helioseal F: There was significant difference at all the intervals except baseline to 1 month and 2 weeks to 1 month. Teethmate F1: There was significant difference at all the intervals except baseline to 2 weeks, baseline to 1 month and 2 weeks to 1 month. Fuji IX: There was significant difference at all the intervals except baseline to 1 month. Ketac Molar: There was significant difference at all the intervals except baseline to 1 month.

**Figure 1:**
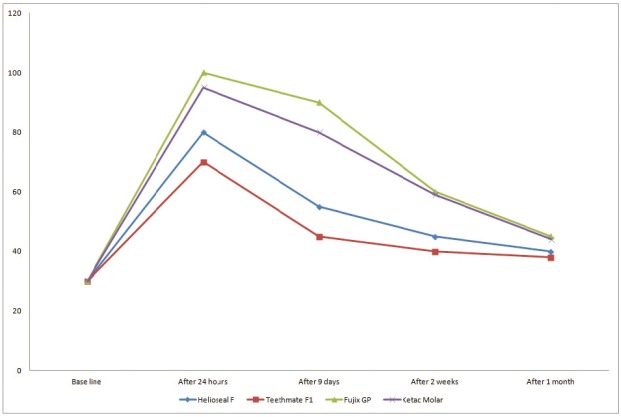
Mean plaque fluoride levels of different pit and fissure sealants at various intervals of time.

Figure 1 depict mean plaque fluoride levels of all the test samples at various intervals of time.

## Discussion

The combination of sealant and fluoride is expected to be additive in preventing dental caries. It will be more beneficial if sealant can increase the fluoride level in the oral environment.

In vitro and in vivo studies (12) have shown that low fluoride levels in the fluid phase surrounding the teeth have a substantial cariostatic potential by inhibiting demineralization and enhancing remineralization.

Studies have shown elevated plaque fluoride concentrations will reduce bacterial acid production (13,14). In our study, an increased dental plaque fluoride levels has been observed, which is similar to the in vitro studies (15-17) and to a study where increase in the fluoride content of plaque were the plaque was grown in a tunnel inside the glass ionomer material (18).

Where as the findings of the present study was in contrast to a vivo study (19) where there was no significant level of fluoride concentrations of enamel and plaque following glass ionomer application.

All the fissure sealants evaluated in this study released measurable amounts of fluoride. This observation is consistent with the findings of many other authors (20-25). Although there was a great quantitative differences in fluoride release among the materials, the rate of fluoride release by the materials were similar.

In Glass Ionomer Cements, there is an acid base reaction resulting in the leaching of Ca2+, Al3+ and F- ions to form a polysalt matrix. This may be responsible for the short term elution process. In composites, there is no acid – base reaction; the only source of fluoride would come from glass filler particles, resulting in a slow diffusive release.

Still the mechanism of fluoride release from fluoride fissure sealants remains speculative. Release might occur from the insoluble sealant material as a result of porosity. It might also occur because the fluoride ion or the fluoride – glass is not tightly bound to the polymerized resin molecules. Unpolymerized resin probably would not be of benefit to the enamel, in the clinical situation because it contacts the enamel only minimally and also would be worn away almost immediately after sealant placement.

Despite the diversity in the reported amount of fluoride release from glass ionomer, present study does share a common finding. The pattern of fluoride release remained consistent, with an initial burst of fluoride release, followed by prolonged leakage. This pattern of fluoride release was observed in previous studies (26-28) and suggests that fluoride release occurs as two different processes, one short term and rapid and the other more gradual and prolonged (28,29).

In the current study, the ability of the examined materials to uptake fluoride from their surroundings was not investigated. It has been proven that all glass ionomer formulations act as rechargeable, slow fluoride release systems after exposure to fluoride solutions such as toothpaste and fluoride rinses (30). This may be clinically important because glass ionomer restorations may act as intraoral devices for the controlled slow release of fluoride at sites at risk for recurrent caries. Composites and compomers however, do not seem to have this ability.

## Conclusions

Fluoride release occurred from all of the materials evaluated over a 4-week period. There was considerable quantitative variation but the pattern of release from the materials was similar showing an initial rapid followed by a slow and long lasting pattern of release. Release peaked with in the first few days of the material being placed in the oral cavity. Fluoride released by the glass ionomer cements were more when compared to methacrylate fissure sealants. Increase in the salivary fluoride level was very low when compared to plaque fluoride levels. Salivary fluoride levels reached almost to baseline values within 1 month of the study and the levels were not statistically significant. Plaque fluoride levels were maintained at a higher level even after 1 month of the study and the levels were statistically significant. As it is unknown that the exact amount of fluoride necessary to inhibit demineralization and enhance remineralization, further studies are necessary to know the same both in saliva and dental plaque.
